# Sn-Doping and Li_2_SnO_3_ Nano-Coating Layer Co-Modified LiNi_0.5_Co_0.2_Mn_0.3_O_2_ with Improved Cycle Stability at 4.6 V Cut-off Voltage

**DOI:** 10.3390/nano10050868

**Published:** 2020-04-30

**Authors:** Huali Zhu, Rui Shen, Yiwei Tang, Xiaoyan Yan, Jun Liu, Liubin Song, Zhiqiang Fan, Shilin Zheng, Zhaoyong Chen

**Affiliations:** 1 School of Physics and Electronic Science, Changsha University of Science and Technology, Changsha 410114, China; juliezhu2005@126.com (H.Z.); 18271692161@163.com (R.S.); zqfan@csust.edu.cn (Z.F.); 2 Qinyuan Jiazhi Institute Co. Ltd., Qingyuan 511517, China; tangyiwei@jianae.com (Y.T.); zhengshilin@jianae.com (S.Z.); 3 School of Materials Science and Engineering, Changsha University of Science and Technology, Changsha 410114, China; richardhipower@126.com (X.Y.); liujun@stu.csust.edu.cn (J.L.); 4 School of Chemistry and Food Engineering, Changsha University of Science and Technology, Changsha 410114, China; liubinsong1981@126.com

**Keywords:** lithium-ion batteries, cathode material, LiNi_0.5_Co_0.2_Mn_0.3_O_2_, Sn-modification, high cut-off voltage

## Abstract

Nickel-rich layered LiNi_1−*x*−*y*_Co*_x_*Mn*_y_*O_2_ (LiMO_2_) is widely investigated as a promising cathode material for advanced lithium-ion batteries used in electric vehicles, and a much higher energy density in higher cut-off voltage is emergent for long driving range. However, during extensive cycling when charged to higher voltage, the battery exhibits severe capacity fading and obvious structural collapse, which leads to poor cycle stability. Herein, Sn-doping and in situ formed Li_2_SnO_3_ nano-coating layer co-modified spherical-like LiNi_0.5_Co_0.2_Mn_0.3_O_2_ samples were successfully prepared using a facile molten salt method and demonstrated excellent cyclic properties and high-rate capabilities. The transition metal site was expected to be substituted by Sn in this study. The original crystal structures of the layered materials were influenced by Sn-doping. Sn not only entered into the crystal lattice of LiNi_0.5_Co_0.2_Mn_0.3_O_2_, but also formed Li^+^-conductive Li_2_SnO_3_ on the surface. Sn-doping and Li_2_SnO_3_ coating layer co-modification are helpful to optimize the ratio of Ni^2+^ and Ni^3+^, and to improve the conductivity of the cathode. The reversible capacity and rate capability of the cathode are improved by Sn-modification. The 3 mol% Sn-modified LiNi_0.5_Co_0.2_Mn_0.3_O_2_ sample maintained the reversible capacity of 146.8 mAh g^−1^ at 5C, corresponding to 75.8% of its low-rate capacity (0.1C, 193.7mAh g^−1^) and kept the reversible capacity of 157.3 mAh g^−1^ with 88.4% capacity retention after 100 charge and discharge cycles at 1C rate between 2.7 and 4.6 V, showing the improved electrochemical property.

## 1. Introduction

The lithium-ion battery (LIB) is one of the most promising power supply devices for portable electronic products and electric vehicles because of its high energy density and power density, long cycle lifetime and environmental benignity among various novel battery systems [1,2,3,4,5]. Because the commercialized LiCoO_2_ cathode material has the disadvantages of high cost and poor thermal safety, the aim of present research work is to develop the prospective alternatives for LiCoO_2_ toward better lithium batteries [6,7,8,9,10]. Nickel-rich layered LiMO_2_ is an important cathode material for LIB because of its superior theoretical discharge capacity compared with that of olivine or spinel materials. Herein, high nickel content in LiMO_2_ is beneficial to increase capacity, high manganese content enhances the structural stability and high cobalt content improves the rate performance [11]. Among these LiMO_2_ materials, LiNi_0.5_Co_0.2_Mn_0.3_O_2_ has been gradually used as a component of commercial lithium secondary batteries due to its low price, high capacity and improved safety. Nevertheless, LiNi_0.5_Co_0.2_Mn_0.3_O_2_ still suffers from several issues, including severe capacity degradation and limited high-rate capability, especially at high cut-off voltage, which is ascribed to the transition metal dissolution and surface structure transformation during cycling [11].

At present, surface coating has been implemented to improve the cyclic property of cathode materials. Coatings like metal oxides (such as Al_2_O_3_ [12,13], antimony doped tin oxide (ATO) [14], CeO_2_ [15], CuO [16], Cr_8_O_21_ [17], MoO_3_ [18], SiO_2_ [19], TiO_2_ [20], ZnO [21], and ZrO_2_ [22]), fluoride (AlF_3_ [23]), lithium salts (such as LiAlO_2_ [24], LiBO_2_ [25], Li_2_MnO_3_ [26], Li_2_MoO_4_ [27], Li_2_SiO_3_ [28], Li_2_TiO_3_ [29], Li_3_VO_4_ [30], and Li_2_ZrO_3_ [31]), and others (polypyrrole (PPy) [32], carbon nanotube (CNT) [33]) are proven to be effective for alleviating the transition metal dissolution and then improving the cyclic property of the cathode materials. On the other side, partial substitution with cations or anions is considered as a promising method to stabilize the crystalline structure of LiNi_0.5_Co_0.2_Mn_0.3_O_2_ materials and improve its high-rate capability, such as bulk doping with Al [24,34,35], K [36], Mo [37], Na [38,39], Nd [40], Ti [41,42], Zr [22,32,42], Y [43], F [44], and Cl [36]. In principle, researchers choose the ions which show a large ionic radius and high electronegativity to substitute for the transition metals in LiMO_2_ because these kinds of ions can expand the channel-like Li^+^ diffusion pathway and decrease the covalence characteristics of cation–oxygen bonds of LiMO_2_ materials. Ion substitution inhibits the release of oxygen and has little effect on the structure of LiNi_0.5_Co_0.2_Mn_0.3_O_2_ materials, so the host lattice of the materials will be well maintained. As we know, the electrochemical properties of LiMO_2_ materials can be greatly enhanced when combined with the advantages of doping and coating co-modification by LiAlO_2_-coating layer and Al-dopant [24]. Mo-coating and doping for LiNi_0.5_Co_0.2_Mn_0.3_O_2_ [45] have been studied by researchers and demonstrated enhanced electrochemical properties.

Among various doping cations, Sn^4+^ has the same ionic radius of 0.69 Å as Ni^2+^, close to that of Li^+^ (0.76 Å). Meanwhile, the bonding energy of Sn-O is 548 kJ mol^−1^, while those of Ni-O, Co-O and Mn-O are 391.6, 368 and 402 kJ mol^−1^, respectively. The high bonding energy of Sn-O is favorable to improve the crystalline structural stability of cathode materials. In addition, Sn^4+^ has a high electronegativity, leading to strong ionicity of the metal–oxygen bond [46]. Therefore, Sn-modification is expected to enhance the cyclic property and high-rate capability of LiMO_2_ materials. The rate performances of some layered LiMO_2_ materials have been enhanced by substituting stannum for transition metals. For example, LiNi_3/8_Co_2/8_/Mn_3/8-*x*_Sn*_x_*O_2_ has enhanced the chemical diffusion coefficient *D*_Li_ of Li-ion, leading to improved rate capability [47]. Sn-doped LiNi_0.8_Co_0.2_O_2_ has increased electronic conductivity because a free electron was released into the conduction band after doping [48]. The electrochemical properties of LiNi_0.8_Co_0.1_Mn_0.1_O_2_ have been enhanced by SnO_2_ at high voltage [49].

In this study, we report a facile synthesis of Sn-doping and Li_2_SnO_3_ in situ coating layer co-modified (Sn-modified) LiNi_0.5_Co_0.2_Mn_0.3_O_2_. The crystalline structures, morphologies, surface chemical states of cations and electrochemical performances of Sn-modified LiNi_0.5_Co_0.2_Mn_0.3_O_2_ samples are characterized. As expected, the amount of Sn has a major impact on the modification treatment. Substituting a large amount of Sn for transition metals in LiNi_0.5_Co_0.2_Mn_0.3_O_2_ can form Li_2_SnO_3_ on the surface. Suitable Sn-substituting can relieve the cation mixing degree and provide a stable structure as well as form the Li^+^-conductive coating layer on the surface of the sample, leading to improved physical and electrochemical properties.

## 2. Materials and Methods

### 2.1. Materials Preparation

Layered Sn-modified LiNi_0.5_Co_0.2_Mn_0.3_O_2_ samples were synthesized using a facile molten salt method. Molten salt (0.76LiOH·H_2_O–0.12Li_2_CO_3_), commercial Ni_0.5_Co_0.2_Mn_0.3_(OH)_2_ precursors and nano-sized Sn powder were used as raw materials and mixed completely by mortar and pestle with the appropriate amount of ethyl alcohol. LiOH·H_2_O with a purity of 98% was bought from Xilong Chemical Co., Ltd. (Shantou, China). Li_2_CO_3_ with a purity of 99.5% was provided by Sichuan Tianqi Lithium Co., Ltd (Chengdu, China). Ni_0.5_Co_0.2_Mn_0.3_(OH)_2_ with a transition metal element content of 62.27% was purchased from Chongqing Teri battery materials Co., Ltd. (Chongqing, China). Sn powder with a purity of 99.9% was bought from Shanghai Chaowei Nano Technology Co., Ltd. The molar ratio of the Li and M in LiMO_2_ was 1.05:1. The mixture was pre-heated at 480 °C for 120 min followed by calcination at 880 °C for 720 min in air atmosphere. Finally, the obtained samples were ground for 30 min for physical and electrochemical property tests. Here, Sn-modified LiNi_0.5_Co_0.2_Mn_0.3_O_2_ compounds, in which certain amounts of transition metals were substituted by Sn, were marked as MS1 (1 mol% Sn), MS3 (3 mol% Sn) and MS5 (5 mol% Sn), and were prepared via the above-mentioned approaches. The pristine LiNi_0.5_Co_0.2_Mn_0.3_O_2_ compounds were obtained through the same method and labeled as M523.

### 2.2. Characterizations 

The crystalline structures of synthesized LiNi_0.5_Co_0.2_Mn_0.3_O_2_ materials were characterized using X-ray diffraction (XRD, D/Max 2000/PC, Rigaku, Tokyo, Japan) with Cu Kα radiation (*λ* = 1.54056 Å) from 10° to 90° with a scan rate of 5° per min. The morphologies of modified samples were characterized by scanning electron microscopy (SEM, Sirion200, FEI Ltd., Eindhoven, The Netherlands). The microstructure of the sample surface was analyzed using transmission electron microscopy (TEM, TECNAI G2 F20, FEI Company, Hillsboro, USA). The element distributions were determined using energy dispersive X-ray spectroscopy (EDS, Model 7426, Oxford, UK). The surfaces of the samples were examined using X-ray photoelectron spectroscopy (XPS, K-Alpha 1063, Thermo Fisher Scientific, Waltham, MA, USA) with AlK*α* line (1486.6 eV) as the source of X-ray. 

The CR2025 cell assembly process, the electrochemical charge and discharge tests and the electrochemical impedance spectroscopy (EIS) tests were conducted according to the experimental section of our recently published article [36].

## 3. Results and Discussion

**Figure 1:**
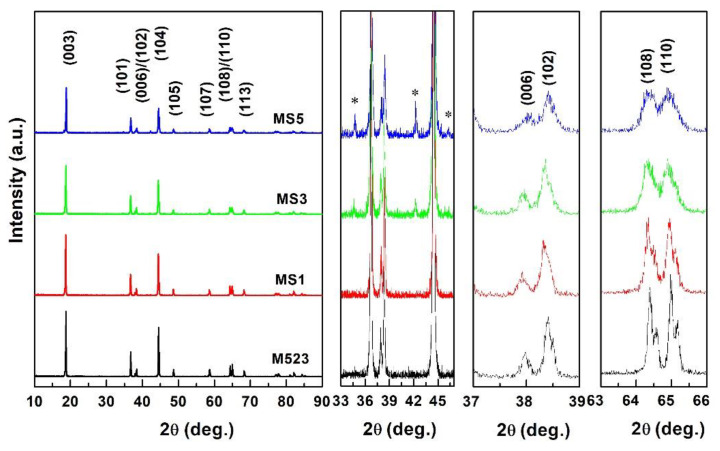
XRD patterns of Sn-modified LiNi_0.5_Co_0.2_Mn_0.3_O_2_ samples (∗ represents Li_2_SnO_3_).

The crystalline structures of the Sn-modified LiNi_0.5_Co_0.2_Mn_0.3_O_2_ samples were studied using XRD in order to characterize the effects of Sn-substitution on the crystal, and the typical diffraction patterns of all samples are demonstrated in Figure 1. The XRD patterns of well-crystallized pristine and Sn-modified LiNi_0.5_Co_0.2_Mn_0.3_O_2_ samples were all indexed to a hexagonal *α*-NaFeO_2_ layered structure (*R*-3*m* space group) with sharp and clear diffraction peaks. The obvious splitting of diffraction peaks of (006)/(102) and (108)/(110) reflects the highly ordered hexagonal structure. However, there are some impurities in the patterns of samples MS3 and MS5 near the 2*θ* of 35 and 43°, which are identified as Li_2_SnO_3_. It is obvious that the formation of Li_2_SnO_3_ phase is related to the amount of dopant. To identify the effects of tin substitution on the structures of Sn-modified samples, the crystallographic data of samples are demonstrated in Table 1. Even though the doping amount was small, the cell parameters of all samples changed, showing that Sn-modification affected the main structure of the host. All the crystallographic data changed, which suggests that the substituting element entered into the crystal lattice. All the crystallographic data ratios c/a are higher than 4.899, showing the highly ordered crystal structure. The I_003_/I_104_ ratios (*R*) of modified samples are larger than the value of 1.2, indicating that Sn-substituting can relieve the cation mixing degree. That is to say, Sn^4+^ helps to stabilize the crystal structure of LiNi_0.5_Co_0.2_Mn_0.3_O_2_ during the Li^+^ intercalation and de-intercalation process because Sn-O has a higher bonding energy than those of transition metals and oxygen. The 3 mol% substituting sample showed the largest intensity ratio *R*’((I_006_+ I_102_)/I_101_) and crystal volume, which may have resulted in the best electrochemical performance.

**Figure 2:**
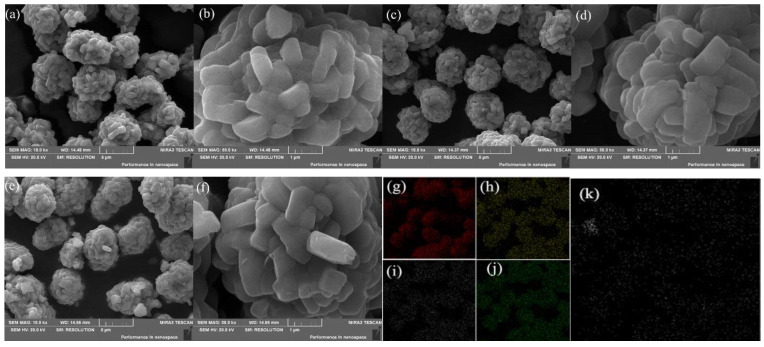
SEM images of MS1 (**a**,**b**), MS3 (**c**,**d**), MS5 (**e**,**f**), and the corresponding EDS mappings of image MS3 (**c**): (**g**)-O, (**h**)-Ni, (**i**)-Co, (**j**)-Mn, (**k**)-Sn.

The SEM images of Sn-modified LiNi_0.5_Co_0.2_Mn_0.3_O_2_ samples and the EDS images are displayed in Figure 2. As is shown, there were no significant differences in the grain sizes from the pristine and Sn-modified samples. All the compounds showed a spherical-like morphology with a particle size from 4 to 6 μm, which is made up by lots of fine primary particles with a length range of 0.5–1 μm. The sample surface was not only compact but also provided enough surface area to make full contact between the cathode and the electrolyte. According to the EDS measurements showing in Figure 2k, it can be obviously seen that stannum and transition metals were uniformly distributed on the surface of the MS3 compound.

**Figure 3:**
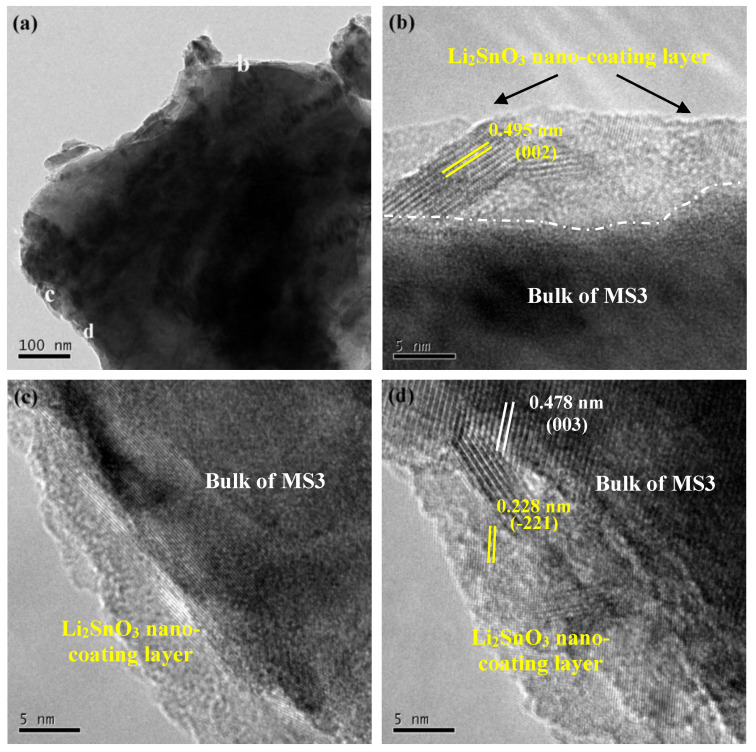
TEM images of MS3 (**a**) and the selected areas in Figure 3a (**b**–**d**).

To reveal the in situ formation of the Li_2_SnO_3_ on the surface of the samples, the microstructure of Sn-modified LiNi_0.5_Co_0.2_Mn_0.3_O_2_ sample MS3 was examined using TEM as shown in Figure 3. It can be seen from Figure 3a that a nano-sized coating layer was obtained on the particle surface of MS3. Three of the coating sites (Figure 3b–d) were enlarged in order to observe the thin layer more clearly. The surface coating layer, which uniformly adhered to the bulk of MS3 particles, had a thickness within the range of 5–10 nm. In addition, Figure 3b,c clearly indicates the crystalline interplanar spacing of 0.495 and 0.228 nm, which can be indexed as (002) and (–221) facets of Li_2_SnO_3_ (JCPDS no. 31-0761), respectively. The (003) facet of bulk MS3 with the crystalline interplanar spacing of 0.478 nm is demonstrated in Figure 3d. These results were consistent with the XRD result and further identified the formation of Li_2_SnO_3_ nano-coating layer.

**Figure 4:**
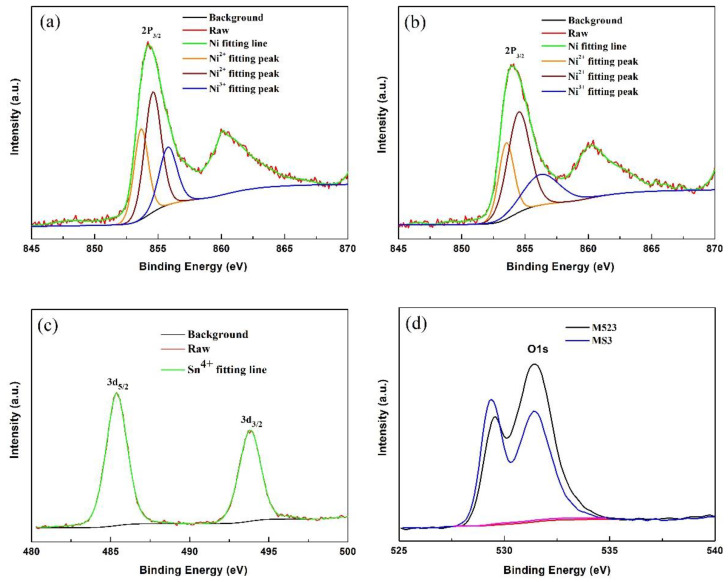
XPS spectra of the synthesized materials: Ni 2p_3/2_ (**a**) spectra of M523, Ni 2p_3/2_ (**b**), Sn3d (**c**) spectra of MS3, O1s (**d**) spectra of M523 and MS3.

To understand the surface chemical composition of the transition metal elements (Ni, Co, Mn) and Sn, the pristine M523 and Sn-modified sample MS3 were examined using XPS. The XPS patterns are shown in Figure 4 and Figure S1. Compared to the pristine M523, the peak positions of Co 2p_3/2_ and Mn 2p_3/2_ in 3 mol% Sn-modified sample MS3 had no obvious shift, showing that the surface chemical states of the transition metals did not change. To further clarify the effect of Sn-modification on the chemical states of cations, the peak positions and mole fractions of transition metal ions and Sn^4+^ in the crystal of M523 and MS3 compounds deduced from XPS fittings are listed in Table 2. According to the corresponding binding energies of Ni 2p_3/2_, Co 2p_3/2_ and Mn 2p_3/2_, we can ascertain that the chemical valences of Ni are Ni^2+^ (853.6 and 854.7 eV) and Ni^3+^(856.2 eV), while those of Co and Mn are Co^3+^ (779.8 eV) and Mn^4+^ (642.4 eV), respectively. The results show that the oxidation valences of Ni, Co and Mn in the Sn-modified samples are still the same as those of the pristine one, only that the mole fraction ratio of Ni^2+^/Ni^3+^ increased from 72.27%/27.73% to 74.88%/25.12% after Sn-modification, which indicates that MS3 has better structure stability. Sn3d peaks appear at 486.5 and 494.9 eV, showing that Sn exists in +4 chemical state [47]. Additionally, Sn-modification has a great influence on the chemical state of O1s. The peak at 529.35 eV was caused by the interaction of transition metal ions and oxygen in the crystal structure, and the peak at 531.54 eV is related to formation of lithium carbonate at the sample surface [50]. The peak intensities of the two characteristic peaks of O1s occur in deflection, which indicates that the lattice oxygen increased and the adsorbed oxygen on the surface decreased after modification. It is beneficial to keep the layered structure stable and reduce the formation of impurities on the sample surface.

**Figure 5:**
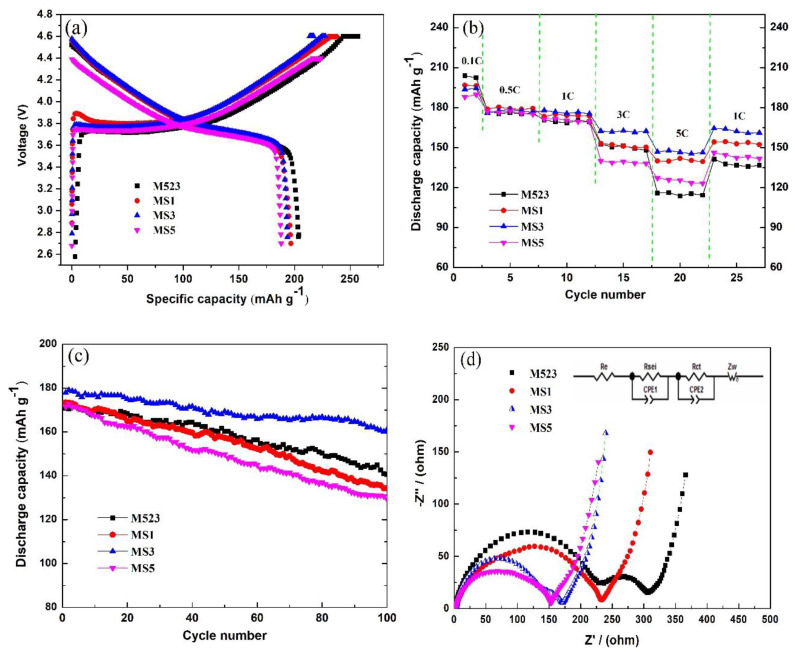
Electrochemical performance of Sn-modified LiNi_0.5_Co_0.2_Mn_0.3_O_2_ samples: (**a**) Initial charge–discharge curves at 0.1C, (**b**) rate performance from 0.1 to 5C, (**c**) cyclic ability at 1C, and (**d**) electrochemical impedance spectroscopy (EIS) plots of Sn-modified LiNi_0.5_Co_0.2_Mn_0.3_O_2_ samples after the 100th cycle.

The curves of electrochemical performance are characterized in Figure 5. The initial charge and discharge capacities were tested at 0.1 C rate at room temperature. We can determine from Figure 5a that the initial discharge capacities for the M523, MS1, MS3 and MS5 samples are 203.9, 196.8, 193.7 and 188.0 mAh g^−1^, and the corresponding coulombic efficiencies are 79.2%, 82.6%, 84.9% and 84.0%, respectively. As discussed previously, a large amount of Sn-doping and Li_2_SnO_3_ impurity existed in MS5, which probably led to the lowest initial discharge capacity. The coulombic efficiency values of the Sn-modified samples are all higher than that of the pristine one. This should be attributed to the Sn-substituting, which can relieve the cation mixing degree and is favorable for Li^+^ transfer. 

The rate performances of LiNi_0.5_Co_0.2_Mn_0.3_O_2_ samples are compared in Figure 5b, in which the charge–discharge cycle was successively taken from 0.1 to 5 C at 2.7–4.6 V for every five cycles. The Sn-modified samples displayed more enhanced rate performance than the pristine M523 at high rates. The MS3 sample presented a reversible capacity of 146.8 mAh g^−1^ at 5 C, corresponding to 75.8% of its initial capacity (0.1C, 193.7mAh g^−1^). However, the pristine M523 kept a reversible capacity of 116.0 mAh g^−1^, just 56.9% of its initial capacity (0.1C, 203.9 mAh g^−1^). This can be attributed to the fact that the bonding energy of Sn–O is higher than those of the transition metal and oxygen in LiNi_0.5_Co_0.2_Mn_0.3_O_2_ samples. It can be seen from the previous XRD results that Sn-substituting can relieve the cation mixing degree and benefit the Li^+^ intercalation/de-intercalation, even in high current density. Furthermore, the formed Li^+^-conductive Li_2_SnO_3_ nano-coating layer prevents the side reaction at the cathode and the electrolyte interface and accelerates the transport of lithium ions as well.

The cyclic stability properties of Sn-modified LiNi_0.5_Co_0.2_Mn_0.3_O_2_ at 1C are illustrated in Figure 5c. It is observed that the Sn-modified sample MS3 exhibited excellent capacity retention with a capacity of 157.3mAh g^−1^ and discharge capacity retention of 88.4% at the 100th cycle, while the pristine sample M523 only kept a capacity of 124.9 mAh g^−1^ and discharge capacity retention of 73.2%. These results indicate that Sn-modification is favorable for keeping the structural stability of the pristine materials and obtaining enhanced cycle performance. Doping can improve the conductivity of the material, and the increase of conductivity after a small amount of doping is reflected in the increase of capacity; however, with the increase of doping amount, the active material decreases, resulting in the loss of electrochemical capacity. Therefore, there is a lack of continuous changes for data shown in Figure 5b,c with the trend of MS5 < MS3 < MS1 or MS1 < MS3 < MS5.

To better understand the effect of Sn-modification on the electrochemical properties of cathode materials, EIS analysis was carried out. Figure 5d demonstrates the EIS profiles of the Sn-modified LiNi_0.5_Co_0.2_Mn_0.3_O_2_ cathodes after the 100th cycle at 1C. According to the equivalent circuit [40] in the inset in which *R*_sei_ stands for the resistance of solid electrolyte interface (SEI) layer, *R*_ct_ is the charge transfer impedance at the interface of electrolyte–electrode, and *Z*_w_ is the Li ion diffusion in the crystal lattice, the EIS spectra were analyzed using Zview-2 software. The values of the total resistance of the *R*_sei_ and *R*_ct_ are 306.0, 233.1, 171.0 and 152.6 Ω for M523, MS1, MS3 and MS5, respectively. It is shown that the *R*_sei_ and *R*_ct_ of the modified samples are significantly smaller than those of the pristine one. The MS3 and MS5 samples show lower total resistance, which may be relative to the formed Li^+^-conductive Li_2_SnO_3_. This is helpful for the intercalation/de-intercalation of Li ions during the charge/discharge process. To investigate the influence of bulk performance of LiNi_0.5_Co_0.2_Mn_0.3_O_2_ modified by Sn-doping on electrochemical performance, the relationships between *ω*^−1/2^ and *Z*’ based on the experimental results are shown in Figure S2. The apparent Li^+^ diffusion coefficient was calculated via a widely used method [8], and it was 1.64 × 10^−10^, 1.75 × 10^−10^ 2.11 × 10^−10^ and 1.82 × 10^−10^ cm^2^ S^−1^ for M523, MS1, MS3 and MS5, respectively. Hence, it could be claimed that Sn-modification contributes to decreasing the charge transfer impedance and improving the Li^+^ diffusion, resulting in better capacity reversibility.

## 4. Conclusions

Uniform near-spherical Sn-doping and Li_2_SnO_3_ co-modified LiNi_0.5_Co_0.2_Mn_0.3_O_2_ were obtained using a facile molten salt method with 0.76LiOH·H_2_O-0.12Li_2_CO_3_, commercial Ni_0.5_Co_0.2_Mn_0.3_(OH)_2_ and Sn nano-powders as the raw materials. The crystal structures, morphologies and electrochemical properties were investigated in detail. The results of the analyses indicate that suitable stannum-modified samples exhibit low cation mixing degrees, enhanced rate capabilities and excellent cyclic performances. Notably, the MS3 sample with 3 mol % Sn-modification aimed at the transition metal site maintained a capacity of 146.8 mAh g^−1^ at the current density of 5C, corresponding to 75.8% of its low rate capacity (0.1C, 193.7mAh g^−1^), while the pristine one kept the capacity of 116.0 mAh g^−1^, just 56.9% of its initial capacity (0.1C, 203.9 mAh g^−1^). The pristine sample also kept the reversible capacity of 157.3 mAh g^−1^ as well as a favorable capacity retention of 88.4% after 100 cycles (2.7–4.6 V, 1C), which is 15.2% higher than that of the pristine M523 (124.9 mAh g^−1^, 73.2%). The MS3 sample exhibited a lower mole fraction of Ni^3+^, implying less structural transition during the charge–discharge cycles. The improvement of the electrochemical properties can be attributed to the suitable Sn-substituting and formed Li^+^-conductive Li_2_SnO_3_, which can relieve the cation mixing degree, offer more stable crystalline structure for the fast Li^+^-intercalation/de-intercalation during repeated cycles and improve the conductivity to obtain enhanced high-rate reversibility and cycle stability. These results illustrate that Sn-modified LiNi_0.5_Co_0.2_Mn_0.3_O_2_ is an excellent cathode material for increasingly wide utilization in the fields of electric vehicles and energy storage systems.

## Figures and Tables

**Table 1:** Lattice constants of Sn-modified LiNi_0.5_Co_0.2_Mn_0.3_O_2_ samples.

Sample	*a* (Å)	*c* (Å)	*c*/*a*	*R*(I_003_/I_104_)	*R*’((I_006_+ I_102_)/I_101_)	V (Å^3^)
M523	2.8673	14.2103	4.956	1.319	0.428	101.18
MS1	2.8694	14.2286	4.959	1.460	0.474	101.46
MS3	2.8710	14.2223	4.954	1.425	0.526	101.53
MS5	2.8715	14.1499	4.928	1.821	0.508	101.04

**Table 2:** Peak positions and mole fractions of the metal elements for M523 and MS3 samples obtained from XPS fittings.

	Sample	Elements					
		Ni^2+^		Ni^3+^	Co^3+^	Mn^4+^	Sn^4+^
Peak position/eV	M523	853.6	854.7	856.2	779.8	642.4	--
	MS3	853.6	854.7	856.2	779.8	642.4	486.4
Mole fraction/%	M523	72.27		27.73	100.0	100.0	--
	MS3	74.88		25.12	100.0	100.0	100.0

## Supplementary Materials

The following are available online at https://www.mdpi.com/2079-4991/10/5/868/s1, Figure S1: XPS spectra of the synthesized materials: Co 2p_3/2_ (a), Mn 2p_3/2_ (c) spectra of M523, Co 2p_3/2_ (b), Mn 2p_3/2_ (d) spectra of MS3; Figure S2: The relationships between *ω*^−1/2^ and *Z*’.
