# Supplementary material for: Sn-Doping and Li2SnO3 Nano-Coating Layer Co-Modified LiNi0.5Co0.2Mn0.3O2 with Improved Cycle Stability at 4.6 V Cut-off Voltage
Source: Nanomaterials (Basel). 2020 Apr 30;10(5):868. doi: 10.3390/nano10050868 (PMC7279306; doi:10.3390/nano10050868)
Supplement: Supplementary file 1 [file nanomaterials-10-00868-s001.pdf]

Supplementary Materials

Sn-Doping and Li_2SnO_3 Nano-Coating Layer Co-Modified $LiNi_{0.5}Co_{0.2}Mn_{0.3}O_2$ with Improved Cycle Stability at 4.6 V Cut-off Voltage

Huali Zhu [1], Rui Shen [1], Yiwei Tang [2], Xiaoyan Yan [3], Jun Liu [3], Liubin Song [4], Zhiqiang Fan [1], Shilin Zheng [2] and Zhaoyong Chen [3,*]

[1] School of Physics and Electronic Science, Changsha University of Science and Technology, Changsha 410114, China; juliezhu2005@126.com (H.Z.); 18271692161@163.com (R.S.); zqfan@csust.edu.cn (Z.F.)
[2] Qinyuan Jiazhi Institute Co. Ltd., Qingyuan, 511517, China; tangyiwei@jianae.com (Y.T.); zhengshilin@jianae.com (S.Z.)
[3] School of Materials Science and Engineering, Changsha University of Science and Technology, Changsha 410114, China; richardhipower@126.com (X.Y.); liujun@stu.csust.edu.cn (J.L.)
[4] School of Chemistry and Food Engineering, Changsha University of Science and Technology, Changsha 410114, China; liubinsong1981@126.com (L.S.)
* Correspondence: chenzhaoyongcioc@126.com

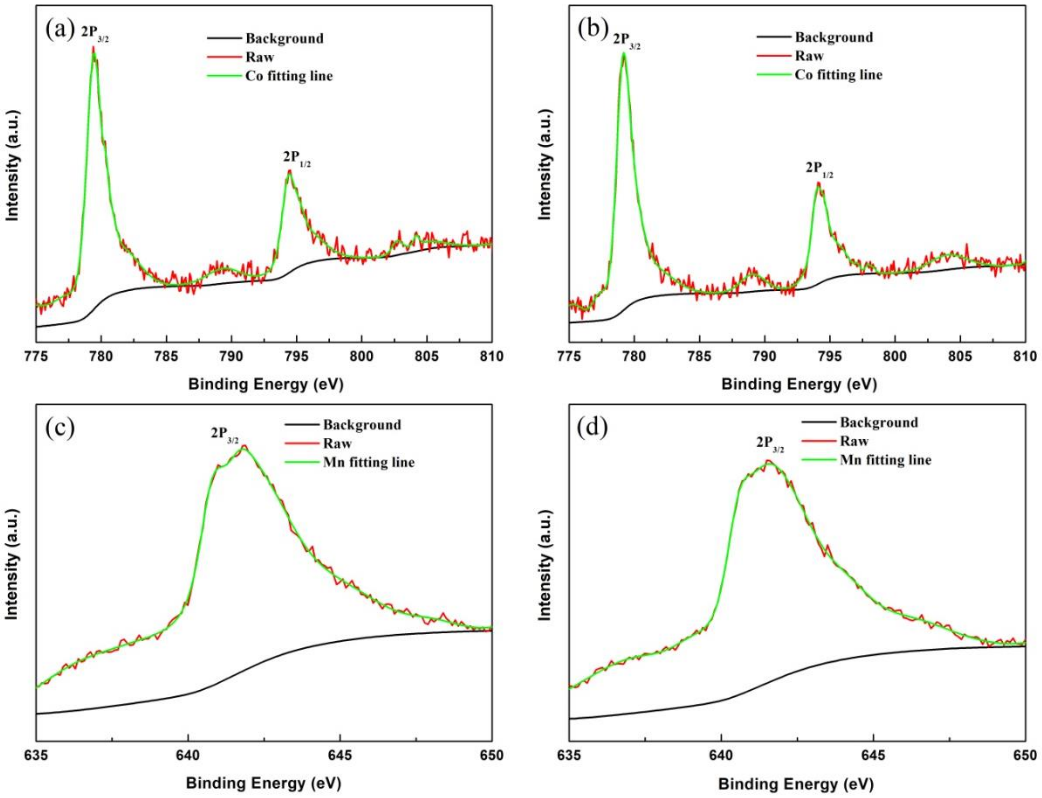

Figure S1. XPS spectra of the synthesized materials: Co $2p_{3/2}$ (a), Mn $2p_{3/2}$ (c) spectra of M523, Co $2p_{3/2}$ (b), Mn $2p_{3/2}$ (d) spectra of MS3.
